# Mr. Ward, on Cancer

**Published:** 1803-11-01

**Authors:** M. Ward

**Affiliations:** Manchester


### *To the Editors of the Medical and Physical Journal.*

GENTLEMEN,

I AM obliged to defer my Remarks on the enclosed Case of Cancer to your next number but one; and probably what I have now sent will occupy as much room as you can conveniently spare in your next.

The objections that have been made to Cullen's definitions of the terms stimulants and sedatives, and no one having thought it worth while to amend them, or to propose others in their stead, or to give their opinion of those proposed by me, (see No. 50, p. 348) have prevented my going on with the subject. I should be sorry for so interesting a topic to be left undecided. Should your Correspondents continue silent, I shall be under the necessity either of proceeding in my own way, or of withdrawing the request I made in your 50th number, p. 335.

I am, &c.

*Manchester, October* 12, 1803. M. WARD.

---

An extraordinary Case of Cancer of the Breast which has lately been under my care, gave rise to the following observations.

THE CASE.

Eliz. Houchard, æt. 58, formerly of a robust healthy constitution, was admitted into the Infirmary, July 12, 1802, with a cancer of the right breast of a year's standing.

The tumour was large, moveable, painful, and slightly ulcerated in the centre: one of the axillary glands was also enlarged, hard, and uneasy.

The breast was in such a state as to admit of extirpation, and had the disease extended no farther, I should not have hesitated to have recommended the operation: it was however a proper case for consultation, the result of which was, an operation, provided the patient should be desirous of having it performed, after the arguments both for and against it had been laid before her.

Being informed it was doubtful what the event of an operation might be, but that it afforded the only chance of a cure, she requested it might be done, which was only prevented from taking place immediately by the continuance of thirst, pain in the loins, and other symptoms of fever, with which she was attacked soon after her admission; and by the time these were subdued, the disease had extended too far to admit of it.

Her spirits now failed, the pain increased, and extended to the arm: in a short time the breast adhered to the pectoral muscles, and began to put on a livid aspect; the superincumbent skin either ulcerated or excoriated, the discharge thin, copious, and extremely offensive.

These changes were soon followed by great debility *and other symptoms of typhus*; and when these had continued some time, she became so delirious and noisy as to make it necessary to remove her into the fever ward. This was about the middle of October,

For several weeks the breast had been growing more and more livid in its appearance, till at length it became completely gangrenous, and by degrees came away, leaving an ulcer of a healthy appearance about as large as a common sized tea saucer.

From that time a great and sudden amendment of all the symptoms was experienced; the fever and pain left her: from a state of the most extreme debility, she recovered her appetite, strength, and spirits surprizingly, and in less than a month returned to the cancer ward in the Infirmary.

 (Impro-

(Improbable as such an event was, she now began to flatter herself with the hope of getting well, which she retained; so entirely did she feel herself relieved in the part originally affected, till within a few days of her death.)

The change in her complexion was very remarkable; and was noticed with surprize by others as well as myself both at that time and in the subsequent part of her illness. The "pale leaden tinge," with which it had been affected, vanished, and her countenance became clear and healthy; the sore looked well; the discharge, from being extremely copious and intolerably offensive, became moderate in quantity, and nearly free from smell; the healing process went on so rapidly, that the ulcer, which after the separation of the mortified parts was at least six inches in diameter, was reduced in about a month to one third of that size; it then remained stationary till the middle of December, when it began to put on a more unfavourable appearance: the newly formed skin began to grow uneven and callous, some parts being rather more prominent than others, and yielding a serous or lymph-like discharge: the axillary gland, which had been easy some time, again became painful, as did also the shoulder and upper part of the arm of the affected side, which on examination were found to be rather swelled; yet her general health did not decline so rapidly as might have been expected.

At length, the whole arm became so much swelled as to render it necessary for her to be almost constantly in a horizontal posture; and independent of the loss of appetite and strength which the want of exercise occasioned, she suffered more pain and inconvenience from this, (reckoning from the time of its attack) than from all her other ailments put together.*

Under all these disadvantages, (to which must be added the increasing discharge from the ulcer on her chest, and a difficulty of breath, with which she was at times affected, from a sense of stricture in the right hypochondrium, during the last two months of her life) she continued till the 2d of May, 1803, having experienced, upon the whole, during the last six months of her confinement, a much greater

* *The progress of the swelling was from above downwards, and the limb was œdematous between the wrist and elbow, but not above. The swelling extended to the scapula*

greater degree of health and spirits as well as freedom from pain, than is usual in the last stage of this deplorable malady.

TREATMENT.

How far the method of treatment adopted, assisted in producing the gangrene and consequent separation of the breast is uncertain; but, doubtless, that event happening was the means of prolonging the patient's life, and to it likewise must be attributed the comparative ease which she afterwards enjoyed.

The operation was relinquished towards the end of August, and I have already described the state of the diseased parts, and of the patient's general health at that time, which were such as to render it highly improbable she could continue long. I then thought it a fair case for experiment; and though it was evidently an incurable one, it was not the less necessary, if any useful inference were to be drawn from the plan pursued, that the treatment should be conformable to rational indications of cure. With this view it appeared requisite, 1st. That the diseased parts should be separated from the sound; (it being impossible to retore their natural actions). 2dly, That the powers of life should be supported.

In pursuing these indications, my first object was, to guard against the necessity of giving frequent and large doses of opium, which would have weakened those powers (namely, the natural, vital, and animal functions), and consequently have retarded instead of promoting what was intended; she was therefore confined to an anodyne at night, (consisting of a grain or a grain and a half of opium) unless when the pain was more severe than common, when the dose was allowed to be repeated once or twice a day; and the object was so far accomplished, that at no time, except in the last week of her confinement, did she take more than four grains and a half in twenty-four hours; in general not more than half that quantity, frequently not more than a fourth; and it relieved her pain, whether situated in the breast, the axilla, or the arm, (when refreshing sleep usually followed) so effectually, and seemed so harmless in other respects, (castor oil or rhubarb being given occasionally, and wine in moderate quanties, either in gruel or negus, sometimes alone, to counteract its sedative properties) that I had no inducement for trying the anodyne effects of the hyoscyamus as a substitute for the opium, as I intended.

 With

With similar views, either diluted nitrous acid, an infusion of chamomile with cinnamon water; compound infusion of gentian; an infusion, or some other preparation of bark, with cinnamon, steel, extract of gentian, &c. was administered throughout, as circumstances indicated. Exercise was also enjoined as far as it could be borne, and the warm bath once or twice a week; but the fears of the patient prevented her from using the latter.

To the parts affected, such applications were made use of as I judged likely to ease pain, such as the ol. camph. applied three times a day for half an hour at a time to the breast, shoulder, and arm pit, and as far round each of them as the pain extended; and ung. cerus. acet. spread on linen as often to the breast, for the purpose of weakening, by its sedative power on the different systems of vessels, the vital principle in that part, and thereby of forwarding the process of separation; for I was afraid of applying caustics to such an extent as would have been required for that purpose.

This was the beginning of September, and about the middle of that month the pain was more severe in the surrounding integuments than in the breast itself, and continued so, at times, as long as she lived. And this was relieved in some degree, for a time, by the tinct. nicot. applied as an embrocation; but it soon lost its power.

As soon as a disposition to gangrene became evident, the bark in substance was given, at short intervals, and was continued in this form as long as any of the mortified parts remained, and some time longer; the quantity of wine was increased to a pint and a half, or two pints in twenty-four hours; an anodyne fomentation, and an ointment consisting of opium, camphire and hog's lard, in different proportions, were applied three times a day. Barley water acidulated, was ordered for common drink, to which wine was sometimes added, and a grain and a half of opium, two or three times in the twenty-four hours. In short, the constitutional treatment during this time was the same as if the mortification had arisen from any other cause.

When it was stopped, a decoction of oak bark with camphorated spirits of wine was ordered as a lotion, and powdered bark to be sprinkled *plentifully* upon the sphacelated parts, two or three times a day, till the whole of the offending matter had been either cut or thrown off.

The parts in the circumference were soonest cleared, and there prepared chalk was applied, instead of the bark, till the whole surface looked clean; after which the ulcer went went on in filling up and contracting rapidly in all directions, as has been described.

To promote this end, various applications were made use of; the chief of which were, a strong decoction or infusion of oak bark,* at first with, afterwards without camphorated spirit of wine;† adhesive plasters, chalk or calamine prepared, cerate of prepared calamine, powdered bark, rhubarb, &c. &c.

When the cicatrix began to ulcerate, powdered savine was applied to the prominent parts, but without effect; pure kali was then applied in its stead, but as it operated merely as a stimulant, without destroying the life of the part, it was discontinued; and from this time the only dressings made use of were, the oak bark lotion, powdered bark, rhubarb, or calamine.‡

About the same time the enlarged axillary gland, which had been quiet so long, again became painful, and contained a small quantity of matter deep seated; and finding it every day grow larger, more painful, and the discolouration of the skin more intense, I ventured to apply pure kali, so as to produce a small eschar; the pain immediately ceased, the swelling and redness subsided; a poultice was then applied till the slough came away, leaving a deep cavity which discharged a curdy matter in small quantity for some time, after which it filled up, skinned over, and continued easy three or four weeks, and then gradually returned to its former state.

Emboldened by the success of the first experiment, I applied a caustic somewhat larger than before, with the same favourable result, and had occasion to repeat it three or four times, and always with advantage. The eschar was never larger than a shilling.

The disease being by these means interrupted in its progress in the breast and axilla, seemed to fall with more violence

---

* A decoction was first used, made by boiling two ounces of oak bark in three pints of water to two pints; and afterwards an infusion, made by infusing four ounces of the former four hours in three pints of boiling water. Towards the last it was made stronger still, viz. four ounces to two pints.

† As long as the camphorated spirit formed a part of the lotion, the sore was flabby and bled frequently; and from the time the infusion of oak bark was used alone, no hæmorrhage occurred, except the part was rubbed or bruised, and it was then very slight and soon ceased.

‡ Perhaps it is unnecessary to mention that warm water was used every morning to the ulcerated parts, and soap occasionally, and that a flannel roller was regularly applied to the arm, from the time it began to swell.

violence upon the lymphatics of the arm, producing the swelling and other effects mentioned above; to alleviate which the following means were made use of: 1st. the vapour bath applied daily; 2d. artificial sea water, with two or three times the usual quantity of salts; 3d. mercurial friction; 4th. dry friction with the hand and flesh brush; 5th. adhesive plasters applied tight from the hand to the shoulder. All these except the 3d, were of use, especially the dry friction and adhesive plasters, which afforded great relief, and continued to do so to the last.

[ To be continued. ]

